# Diagnosis of mycosis fungoides following upadacitinib treatment

**DOI:** 10.1016/j.jdcr.2026.07.064

**Published:** 2026-08-07

**Authors:** Tajena Jones, Ted V. Jacoby, Peggy A. Wu

**Affiliations:** a University of California Davis, School of Medicine, Sacramento, California; b Department of Dermatology, University of California Davis, Sacramento, California

**Keywords:** atopic dermatitis, CTCL, cutaneous T-cell lymphoma, JAK inhibition, JAK inhibitor, mycosis fungoides, upadacitinib

## Introduction

Upadacitinib is an oral Janus kinase (JAK)-1 selective inhibitor approved by the Food and Drug Administration to treat atopic dermatitis (AD).^1^ Adverse effects of JAK inhibitors include upper respiratory infections, nasopharyngitis, herpes outbreaks, nausea, acne, headaches, and potentially increased risk of malignancy.^1^ Data on lymphoma risk from upadacitinib are limited, and it remains unclear whether there is a mechanistic link; yet cases of mycosis fungoides (MF)/cutaneous T-cell lymphoma (CTCL) have been reported after its use for AD, mainly in middle-aged or older adults.^2^^,^^3^ Here, we describe a case of CTCL diagnosed after the use of upadacitinib for the treatment of AD in a young adult.

## Case presentation

**Fig 1:**
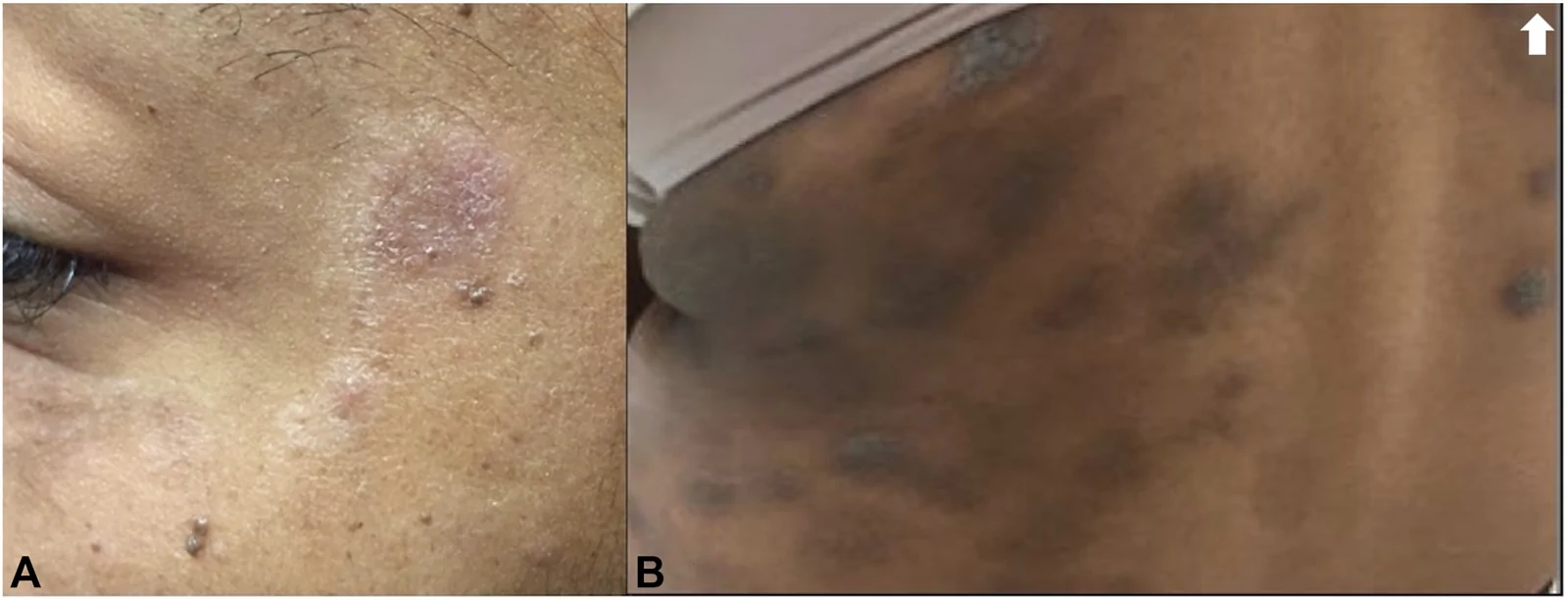
**A,** Baseline AD 5 years prior to upadacitinib and 3 years prior to any systemic treatment, plaque with scale in left temporal region. **B,** Baseline AD 5 years prior to upadacitinib and 3 years prior to any systemic treatment, large brown plaques on upper to mid back.

**Fig 2:**
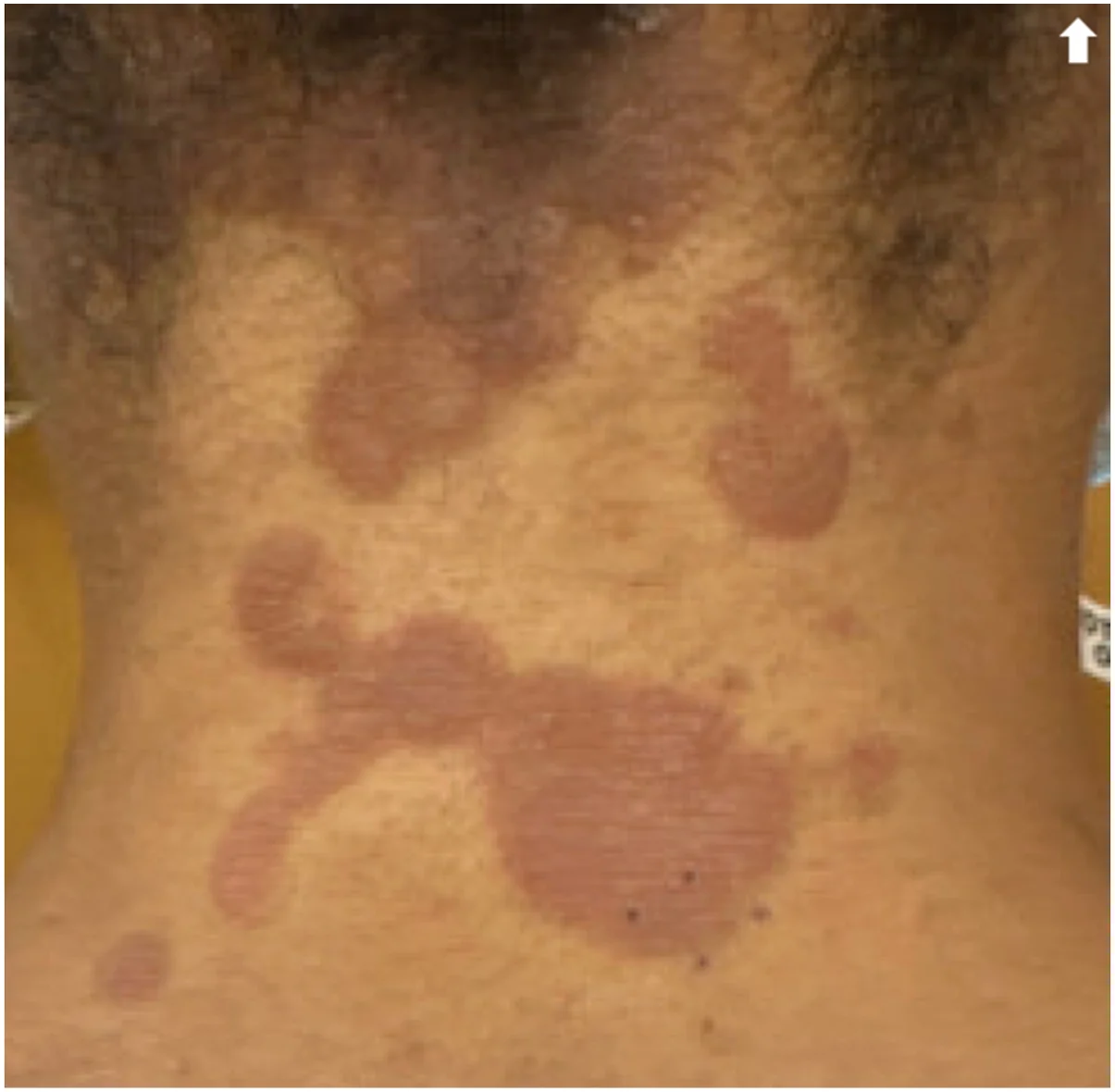
Neck at time of second biopsy consistent with AD diagnosis 1 year prior to upadacitinib.

A 29-year-old woman with a 9-year history of severe AD presented with a progressing eruption of violaceous plaques 2 weeks after starting upadacitinib. The patient did not have a personal or family history of other components of atopy. Initially her skin disease presented as plaques ranging in color from light pink to brown (Fig 1, *A* and *B*). In 2019, 5 years prior to presentation, a punch biopsy of her upper right arm showed spongiotic dermatitis with eosinophils consistent with AD. Her disease was managed with topical corticosteroids (triamcinolone 0.1%, hydrocortisone 2.5%, fluocinonide 0.05%). Additionally, dupilumab 300 mg subcutaneously was started April 2022 and discontinued in October 2022 after lack of improvement. In July 2023, her disease consisted of scaly magenta plaques on her bilateral forearms, anterior neck and posterior neck/scalp, along with scaly, pink patches on her legs bilaterally. A second punch biopsy performed on the neck showed subacute spongiotic dermatitis (Fig 2).

**Fig 3:**
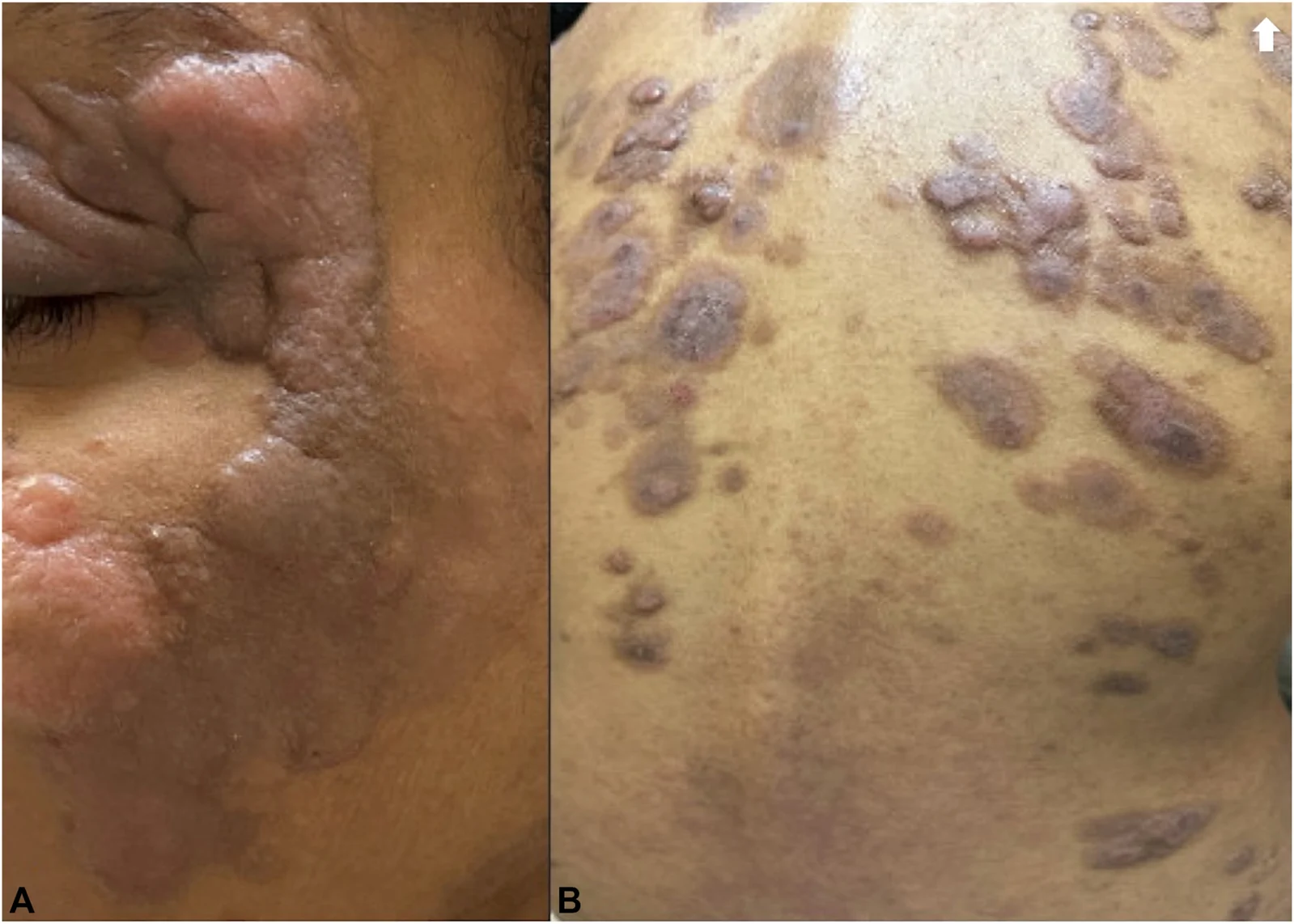
**A,** Firm, violaceous plaques with a dusky center on upper back after upadacitinib. **B,** Violaceous dermal plaques on the forehead, left eye, and left cheek after upadacitinib.

Due to patient relocation, she was not seen by a dermatologist for a year, but meanwhile experienced symptom progression. In September 2024, she started oral upadacitinib 15 mg daily for 9 days before discontinuing for lack of treatment response and worsening of skin disease. Two weeks after discontinuation, she reconnected with dermatology for a skin eruption different from her baseline. Examination of her upper back showed firm, violaceous plaques with a dusky center (Fig 3, *A*). Her lower extremities, lower back, and abdomen displayed light purple to violaceous indurated coalescing papules and plaques. The patient developed violaceous dermal plaques in her eyelids, forehead, and cheeks (Fig 3, *B*). Hematoxylin and eosin staining and immunohistochemistry of punch biopsies from the abdomen and back revealed epidermotropism, a perivascular and interstitial infiltrate of small to large lymphocytes in the dermis staining positive for CD3, CD2, CD5, CD4, CD30, and dim to negative for CD7, CD26, CD8, positive TCR-beta, negative TCR-gamma, consistent with MF meeting criteria for large cell transformation. Blood flow cytometry did not reveal significant involvement of atypical cells. Although the patient denied having systemic symptoms, whole-body PET/CT revealed increased fluorodeoxyglucose activity in numerous cutaneous lesions and within axillary and inguinal lymph nodes.

She subsequently started systemic brentuximab 1.8 mg/kg every 3 weeks, which was well tolerated except for nausea and vomiting. After her fourth infusion, the patient experienced resolution of lymphadenopathy. Six months after initiating brentuximab, the plaques improved significantly. One year since MF diagnosis, she remains on the brentuximab infusions with a plan for total skin electron beam therapy to consolidate response.

## Discussion

This case highlights a complex interplay between AD inflammation, malignancy, and JAK inhibition. The similarities in clinical morphology can make differentiation of AD and CTCL difficult. AD itself has been associated with higher odds of CTCL development in cohort studies, with increased risk in those with more severe disease.^4^ There may also be an overlap in the pathophysiology of AD and CTCL. Similar to AD, in early MF Th2 cells produce many cytokines (IL-4, IL-5, and IL-13), and this dysregulation of cytokines is thought to enhance tumor progression.^5^ Meanwhile, a phase 2 clinical trial of oral JAK2 inhibitor ruxolitinib 20 mg administered twice daily in adults with peripheral T/natural killer (NK)/CTCL demonstrated antitumor activity against T-cell lymphomas in some patients.^6^

There is no established causal association between systemic therapies and CTCL, and the nature of the relationship between this patient’s AD and subsequent CTCL diagnoses remains unknown—whether it was misclassification, unmasking, or causal. Retrospective case series have reported potential unmasking of CTCL in the setting of immunomodulation.^7^, ^8^, ^9^ One example is of cyclosporine, a calcineurin inhibitor.^7^ Additionally, a systematic review found that while dupilumab, a monoclonal antibody blocking IL-4 and IL-13 signaling, improved symptoms in some patients, others with CTCL experienced disease progression.^9^ In a series of 7 patients who experienced CTCL progression after dupilumab treatment, 4 had a prior diagnosis of AD and 3 were treated off-label for CTCL, which eventually progressed to Sézary syndrome.^8^

To date, 4 other cases of MF have been reported following upadacitinib treatment of AD (Table I).^2^^,^^3^ In these cases, patients ranged from 44-82 years old, and median time to diagnosis after initiation of upadacitinib was 8 months. Bui et al describe a patient with AD resistant to corticosteroids, injected steroids, methotrexate, phototherapy, and 3 months after starting upadacitinib, the patient was diagnosed with Sézary syndrome/MF.^2^ Mo and Friedmann report a man with presumed AD treated with upadacitinib for 7 months.^3^ One month after discontinuing treatment due to infection, he experienced hair loss, B symptoms, pruritus, and he was subsequently diagnosed with pilotropic MF.^3^ Liao et al reports 2 patients treated with both upadacitinib and dupilumab who developed CTCL.^10^

**Table I:** Summary of patients with CTCL after upadacitinib treatment for AD or an eczematous rash

Reference	Number of patients	Age	Sex	Clinical features of presumed AD at presentation	Histopathologic confirmation of AD	Presence of atypical clinical or histologic findings at baseline	Clinical and histologic finding of CTCL at time of diagnosis
Bui et al^2^	1	52	F	Extremity and trunk involvement	Irregular epidermal acanthosis with mild spongiosis	Minimal improvement with methotrexate, topical corticosteroid, intramuscular steroids, and phototherapy	Diffuse exfoliative erythroderma Focal Pautrier microabscesses, intraepidermal atypical cells a subpopulation of CD4+ cells
Mo and Friedmann^3^	1	44	M	Pruritus and erythematosquamous plaques on the patient’s face extremities, and trunk	∗	No previous history of atopy	Hair thinning, leonine facies, weight loss, and night sweats CD4/CD8 ratio of 22:1 and TRBC1 monoclonality
Liao et al^10^	2	62-82	F M	∗	∗	∗	T-cell monoclonality
Present case	1	29	F	Light pink plaque with scale on right temporal region. Brown plaques on dorsal trunk	Spongiotic dermatitis with eosinophils	Minimal improvement with topical corticosteroids, no familial or childhood history or AD, and no presentation of additional components of atopy.	Violaceous dermal plaques on lower extremities, back, abdomen, and face Epidermotropism and a perivascular and interstitial infiltrate of small to large lymphocytes

*CTCL*, Cutaneous T-cell lymphoma; *F*, female; *M*, male; *MF*, mycosis fungoides.

∗ Information was not provided by reference.

We report the diagnosis of large cell-transformed MF in a young patient following treatment with upadacitinib. Limitations in our case include the accessibility of prior diagnostic specimens to make longitudinal comparisons of immunophenotype and clonality. Given that our patient was on upadacitinib for a short period of time and did not have classic AD associations or disease course, it is possible that she had undiagnosed CTCL prior to upadacitinib treatment, and it is unclear if and what interactions occurred with JAK inhibition. We feel this experience, including the atypical history of atopy, lack of disease control, change in morphology that prompted reassessment and subsequent MF diagnosis, is important to share. Providers should consider reevaluation including repeat biopsies with ancillary studies for rashes with atypical morphology or disease course.

## Conflicts of interest

None disclosed.
